# Epidemiological Characteristics and Economic Impact of Lumpy Skin Disease, Sheeppox and Goatpox Among Subsistence Farmers in Northeast Nigeria

**DOI:** 10.3389/fvets.2020.00008

**Published:** 2020-01-29

**Authors:** Georgina Limon, Ahmed A. Gamawa, Ahmed I. Ahmed, Nicholas A. Lyons, Philippa M. Beard

**Affiliations:** ^1^The Pirbright Institute, Woking, United Kingdom; ^2^Bauchi State College of Agriculture, Bauchi, Nigeria; ^3^European Commission for the Control of Foot-and-Mouth Disease, Food and Agriculture Organisation of the United Nations, Rome, Italy; ^4^Infection and Immunity, The Roslin Institute, Edinburgh, United Kingdom

**Keywords:** outbreak investigation, economic impact, capripoxvirus, lumpy skin disease, sheep and goat pox, Nigeria

## Abstract

Lumpy skin disease (LSD), sheeppox (SP), and goatpox (GP) are contagious viral infections, affecting cattle (LSD), sheep and goats (SP and GP) with highly characteristic clinical signs affecting multiple body systems. All three diseases are widely reported to reduce meat, milk, wool and cashmere production although few studies have formally evaluated their economic impact on affected farms. This study aimed to estimate the economic impact and epidemiological parameters of LSD, SP, and GP among backyard and transhumance farmers in northeast Nigeria. A retrospective study was conducted on herds and flocks affected between August 2017 and January 2018 in Bauchi, Nigeria. Herds and flocks were diagnosed based on clinical signs and information was collected once the outbreak concluded using a standardized questionnaire. Data were collected from 99 farmers (87 backyard and 12 transhumance). The median incidence risk and fatality rate were 33 and 0% in cattle, 53 and 34 % in sheep; 50 and 33% in goats, respectively, with young stock having higher incidence risk and fatality rates than adults. Almost all farmers (94%) treated affected animals with antibiotics, spending a median of US$1.96 (min US$0.19–max US$27.5) per herd per day. Slaughtering or selling affected animals at low prices were common coping strategies. Farmers sold live cattle for 47% less than would have been sold if the animal was healthy, while sheep and goats were sold for 58 and 57% less, respectively. Milk production dropped 65% when cows were clinically affected and 35% after they recovered. Cattle lost a median of 10% of their live weight and sheep and goats lost 15%. Overall economic losses at farm level range from US$9.6 to US$6,340 depending on species affected and production system. Most of the farmers (72%) had not replaced all affected animals at the time of the study. Livestock markets were the most common place to sell affected animals and buy replacements, suggesting these are likely hubs for spreading infections. This study confirms the immediate and long-lasting impact of these diseases on subsistence farmers' livelihoods in North-East Nigeria and suggests potential mechanisms for targeted control.

## Introduction

**Figure 1:**
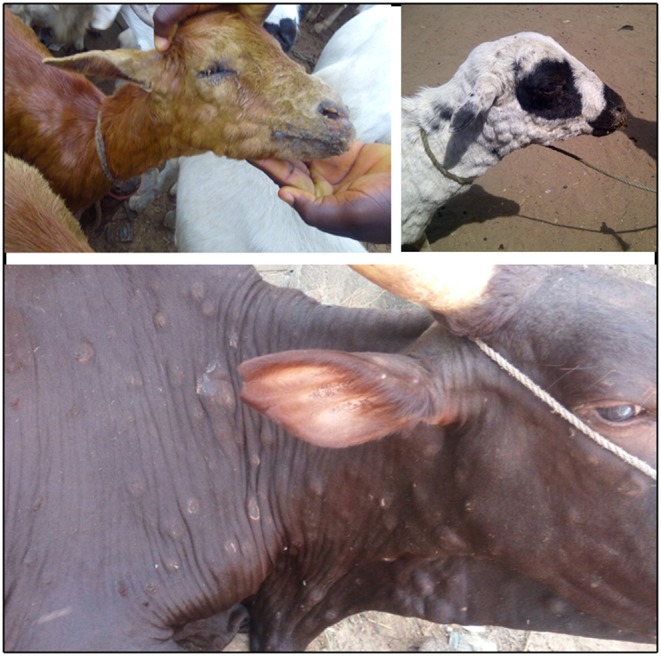
Typical cutaneous papulus observed in goats (top left), sheep (top right) and cattle (bottom) in Bauchi, Nigeria. *Photos credit to AAG*.

The three species of poxvirus in the genus Capripoxvirus (CPPV) are Lumpy skin disease virus (LSDV), Sheeppox virus (SPPV), and Goatpox virus (GTPV). These viruses cause Lumpy skin disease (LSD), sheeppox (SP), and goatpox (GP), three high consequence transboundary diseases capable of causing substantial loss to livestock production systems through morbidity, mortality, enforced control measures, and reduced trade. The three CPPVs cause highly characteristic clinical signs of cutaneous, multifocal to coalescing papules, pustules and nodules of between 0.5 and 3 cm in diameter (Figure 1). In addition, affected animals exhibit weight loss, reduced milk production, depression, lethargy and fever, and in severe cases death (1–5). LSD also reduces the value of hides, while SP and GP can decrease wool and cashmere production (6, 7).

The severity of outbreaks of sheeppox and goatpox varies although in general the morbidity is around 20% and case fatality rate up to 40% (8–11). In contrast LSDV has a lower morbidity of 9–26% and mortality 0.5–2% in non-endemic areas (3, 4, 12–14), compared to a morbidity and mortality of 4.8 and 0.03%, respectively, in endemic areas (15). The three capripoxviruses cause disease only in ruminants and are not zoonotic. LSDV is highly host restricted and affects cattle and water buffalo only, while SPPV and GTPV cause disease in both sheep and goats. Diagnosis of CPPV disease in low and middle income countries is primarily based on clinical signs. Disease confirmation, if required, is usually performed with the polymerase chain reaction (PCR) (16).

Transmission of LSDV, SPV, and GPV is not fully understood, especially in endemic settings. Previous studies suggest that SPV and GPV transmission occurs through aerosol and direct contact, with mechanical transmission by insect vectors playing a minor role. On the contrary, LSDV is believed to be mainly transmitted by blood feeding insects (5, 17–19). Animal movement, assembling of animals from different herds in close contact, and introduction of new animals (without quarantine) in naïve herds have been identified as important risk factors for SP, GP, and LSD (1, 5, 11, 17, 20, 21).

LSD is present in many African and Middle Eastern countries. SP and GP have a wider geographic range and are found in Africa, the Middle East, and Asia including China and Mongolia (5, 7, 18). The CPPV diseases pose an immediate threat to free countries. During 2016–2018, LSD reached countries of Eastern Europe for the first time, with outbreaks reported in Turkey, Greece, Bulgaria, the Republic of North Macedonia, Serbia, Kosovo, Albania, and Montenegro (19, 22). Isolated outbreaks of SP and GP have been reported in Greece, most recently in 2017–2018 (23). Changes in climate conditions, civil unrest in endemic countries and increases in animal movement have raised concerns that the CPPV might keep spreading to countries that were previously free, stressing the urgent need of better understanding the dynamics of these diseases in order to inform policy. Although vaccination with live attenuated strains of CPPV has been shown to be an effective control method in endemic countries (5), prevention and control in free populations is a subject of debate given the impact on trade.

**Figure 2:**
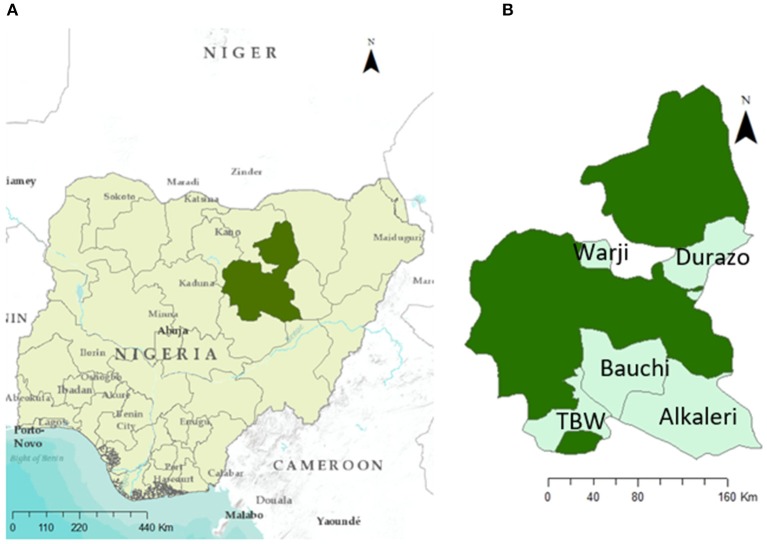
Study location. **(A)** Bauchi State highlighted in dark green and **(B)** local goverments considered in this study within Bauchi State, Nigeria.

Nigeria is located in West Africa bordered by Benin to the West, Niger to the North, Chad to the North East, and Cameroon to the East, with entry to the Gulf of Guinea from the southern part of the country (Figure 2). Most of the cattle (90%), sheep and goat population (70%) of Nigeria are concentrated in the northern region of the country. Bauchi state is located in North-East Nigeria and it is divided in 20 local governments. Bauchi has an estimated livestock population of 3.5 million sheep, 5 million goats and 1.9 million cattle (24). Livestock are mainly kept by subsistence farmers in either pastoralist (transhumance) herders or backyard (sedentary) systems. Based on the experience of the authors, livestock populations tend to increase during the dry season when farmers from drier areas move animals through Bauchi to access better pastures and to be sold in local markets. In addition, it is common practice for middlemen and butchers to purchase live animals in Niger and Chad (where animals tend to be cheaper) and bring them to Nigeria to sell them. There can be a lack of border check points and quarantine controls in place between neighboring countries and Nigeria and empirical observations suggest that previous outbreaks of SP in Bauchi have started following introduction of infected sheep from Niger.

As in most low-middle income countries (LMIC), livestock in Nigeria contribute to farmers' livelihoods through income generation, a direct source of food for home consumption and as part of a coping strategy in emergency situations. LSD, SP, and GP reduce meat and milk production and decrease the value of the animals affected (5), potentially having an important negative impact to farmers' livelihoods. However, the financial impact of CPPV diseases to farmers in Nigeria has not been quantified and coping strategies used by farmers are unknown as for other endemic countries. Currently no official vaccination control programme exists in Nigeria and commercial vaccines are not readily available.

Quantifying the economic impact and epidemiological parameters in endemic areas is critical for informing the design of disease control programmes and allocation of limited resources, as well as improving preparedness in free countries. This study aims to estimate the economic impact and epidemiological parameters of LSD, SP, and GP among affected backyard and transhumance farmers in Bauchi, Nigeria and to identify the main coping strategies followed by farmers that could play a role in the virus transmission.

## Materials and Methods

### Study Design and Data Collection

A retrospective study was conducted on herds and flocks affected from August 2017 to January 2018 using convenience sampling in five local governments of Bauchi State, Nigeria: Alkaleri, Bauchi, Darazo, Tafawa Balewa, and Warji (Figure 2B). Affected herds were identified following report of an outbreak (by the farmer) to an animal health worker or veterinarian. Animal health workers in turn reported the outbreak to the local veterinarian. In addition, further outbreaks were identified by students from Bauchi State College of Agriculture in their villages. All affected herds and flocks were visited following report or identification of an outbreak and diagnosed based on clinical signs (i.e., skin nodules and nasal discharge) by a qualified veterinarian (AAG). Those confirmed and in an accessible location were asked to take part in the study once the outbreak was concluded (i.e., when lesions become necrotic in all animals affected in the herd or flock). For those that accepted to take part, information on morbidity, mortality, changes on production parameters, prices paid for healthy, and affected animals, actions taken toward affected animals and costs incurred was collected by either the local veterinarian or undergraduate students from Bauchi State College of Agriculture once the outbreak concluded. This was conducted using a standardized questionnaire once the outbreak concluded.

Ethical approval was granted from the Directorate of Veterinary Services, Ministry of Agriculture and Natural Resources in Bauchi State Nigeria.

### Data Analysis

Questionnaire data were entered into an Excel spreadsheet by AAG, inconsistences across the data were checked and verified by GL. Descriptive statistics were generated stratified by production type (backyard or transhumance), local government, species (cattle, sheep or goats) and age category (young stock <1 year old and adults ≥1 year old). Parameters estimated include outbreak duration, incidence risk, fatality rate, treatment cost, differences in price between healthy and affected animals sold, place of sales, reduction in milk production (during and after the outbreak), and weight loss.

Outbreak duration was defined as the time period between the date the first animal in the herd or flock presented clinical signs to the date the last animal in the herd recovered or died. Incidence risk was estimated as the number of animals with clinical signs divided by the total animals in the herd or flock at the beginning of the outbreak. Fatality rate was estimated as the number of animals that died divided by the number of animals with clinical signs during the outbreak period. The percentage reduction in animal price and milk yield was calculated comparing the estimated values in diseased and health animals. Changes in price or production were assumed to be only due to the disease.

### Estimation of Total Economic Losses During the Outbreak

**Figure 3:**
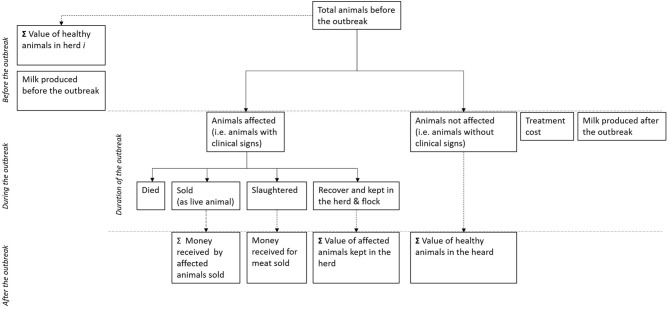
Conceptual framework used to estimate the economic losses caused by Capripox diseases in subsistence farmers in Bauchi, Nigeria.

The conceptual framework to estimate the total economic losses at herd level during the outbreak is presented in Figure 3. The framework is based on the responses given by individual farmers during the survey and the economic loss are therefore estimated at herd level.

### Value of the Herd Before the Outbreak

First the value of the herd or flock before the outbreak was estimated. The herd size before the outbreak equates to the sum of those animals that presented with clinical signs plus those that did not present with clinical signs. We assumed the value of the animals in the herd is the price farmers reported they would get paid if they sold healthy animals. For those farmers that did not report the price of healthy animals, we used the median values reported by those that did report prices. The total value of the herd was then estimated by multiplying the value of the animals by the number of animals stratified by species (Table 1).

**Table 1:** Equations used to estimate the economic losses due to Lumpy skin disease, sheep pox, and goat pox in subsistence producers in Bauchi, Nigeria.

	**Equation used**	**Abbreviations meaning**
Value of the herd before the outbreak	*Vh*_*BeforeOutbreak i*_ =_*P*_*hc i*_ * *Tc*_*i*_+*P*_*hs i*_ * *Ts*_*i*_+ *P*_*hg i*_ * *Tg*_*i*__	P_hc i_, P_hs i_, and P_hg i_ are the prices farmer from herd *i* reported would get paid if they sell healthy cattle, sheep, and goats, respectively Tc *_*i*_* Ts *_*i*_*, and Tg *_*i*_* are the total cattle, sheep and goats in herd *i*
Value of the herd after the outbreak	*VNoAffectedAnimals*_*AfterOutbreak i*_ =_*P*_*hcattle i*_ * *N*_*hcattle i*_+_*P*_*hsheep i*_ * *N*_*hsheep i*_+*Phgoats i*__ * *N*_*hgoats i*_	VNoAffectedAnimals_AfterOutbreak i_ represent the value of animals that did not present clinical signs during the outbreak in herd *i*; P_hcattle i_, P_hsheep i_, P_hgoats i_ price obtained for cattle, sheep, and goats, respectively, without clinical signs (healthy) in herd *i*; N_hcattle i_, N_hsheep i_, and N_hgoats i_ are the number of cattle, sheep, and goats that did not present clinical signs during the outbreak in herd *i*
	*VAffectedAnimalsKept*_*AfterOutbreak i*_ =_*P*_*dcattle i*_ * *N*_*dcattle i*_+_*P*_*dsheep i*_ * *N*_*dsheep i*_+*Pdgoats*__ * *N*_*dgoats i*_	VAffectedAnimalsKept_AfterOutbreak i_ represent the value of animals that presented clinical signs during the outbreak and were kept in herd *i*; P_dcattle i_, P_dsheep i_, P_dgoats i_ price of diseased (i.e., presented clinical signs) cattle, sheep, and goats, respectively, in herd *i*; N_dcattle i_, N_dsheep i_, and N_dgoats i_ are the number of cattle, sheep, and goats that presented clinical signs during the outbreak and were kept in herd *i*
	*MoneyLiveAnimalsSold*_*DuringOutbreak i*_ =_*P*_*dcattle i*_ * *N*_*dcattleSold i*_+_*P*_*dsheep i*_ * *N*_*dsheepSold i*_+*Pdgoats*__	MoneyLiveAnimalsSold_DuringOutbreak i_ represent the money obtained from affected animals sold during in herd *i*; P_dcattle i_, P_dsheep i_, P_dgoats i_ price of diseased (i.e., presented clinical signs) cattle, sheep, and goats, respectively, in herd *i*; N_dcattleSold i_, N_dsheepsold i_, and N_dgoatsSold i_ is the number of cattle, sheep, and goats sold during the outbreak in herd *i*
	*MoneyMeatSold*_*DuringOutbreak i*_ = *Mea*_*t*_*dcattleSold i*_+*Mea*_*t*_*dsheepSold i*_+*MeatdgoatsSold i*__	MoneyMeatSold_DuringOutbreak_ is the money obtained from meat sold after slaughtering animals with clinical signs; Meat_dcattleSold_ *i*, Meat_dcattleSold_ *i*, and Meat_dcattleSold_ *i* represent the money obtained from selling cattle meat, sheep meat, and goat meat, respectively.
	*Vh*_*AfterOutbreak i*_ = *VAffectedAnimalsKep*_*t*_*AfterOutbreak i*_+_*VNoAffectedAnimals*_*AfterOutbreak i*_+*MoneyLiveAnimalsSold*_*DuringOutbreak i*_+*MoneyMeatSold*_*DuringOutbreak i*_	Vh_AfterAutbreak i_ is the totalvalue of the herd after the outbreak
Income loss due to reduced milk production	*Milk*_*IncLoss i*_ = (*MilkYiel*_*d*_*BeforeOutbreak i*_−*MilkYieldDuringOutbreak i*_) * *Outbreak*_*duration i*_ * *Milk*_*price*_	Milk_IncLoss i_ represent the income loss due to milk production in herd *i;* MilkYield_BeforeOutbreak i_ is the average daily milk yield before the outbreak in herd *i*; MilkYield_DuringOutbreak i_ is the average daily milk yield during the outbreak in herd *i*; Outbreakduration *i* is the outbreak duration in herd *i*; Milk_price_ is the average milk price per litter in the study area.
Total economic losses	*Tloss*_*i*_ = (*V*_*h*_*BeforeOutbreak i*_−*VhAfterOutbreak i*_)+*Tx*_*i*_+*Milk*_*IncLoss i*_	Tloss _i_ represent the economic losses in herd or flock *i*; Vh_BeforOutbreak i_ represent the value of herd *i* before the outbreak; Vh_AfterOutbreak_ the value of herd *i* at the end of the outbreak; Tx *_*i*_* is the money spent in treatment in herd *i* and MilkLoss *_*i*_* is the economic losses due to milk loss in herd *i*.

### Value of the Herd After the Outbreak

To estimate the value of the herd or flock after the outbreak, we assumed that animals are either affected (i.e., showed clinical signs) or not affected (i.e., did not show clinical signs). Affected animals can then have four mutually exclusive outcomes (i) died: for which the value of the animal is completely lost (loss due to mortality), (ii) be sold as live animal at a lower value; (iii) be slaughtered and some (or all) of the meat be sold at a lower value, and (iv) be kept in the farm and recover with the value of the animal being lower that would have been if it had not be affected by the disease.

To estimate the money received for animals that were sold as live animals or as meat (following slaughter), as well as the value of animals that presented clinical signs but were kept in the herd or flock, we used the values farmers reported they received when selling animals or meat from animals with clinical signs. For those farmers that kept all animals with clinical signs until they recovered and did not report prices from selling affected animals, we used the median price given by farmers that did sell animals. To estimate the value of the animals that did not present clinical disease we used the value farmers reported would get paid if they sell healthy animals.

The total value of the herd or flock after the outbreak was then estimated as the sum of the value of no affected animals in the herd plus the value of affected animals kept in the herd until recovery, plus the money received by affected animals sold plus the money received for meat sold from affected animals. The equations used are presented in Table 1. As the money obtained by selling animals (live or as meat) is not always re-invested in livestock, we also estimated the value of the herd after the outbreak without considering the money obtained by selling affected animals live or as meat, in other words assuming this money was used for some other expense in the household and was no longer part of the herd value.

### Treatment Cost

Treatment cost during the outbreak equaled the money farmers reported spending for treating affected animals with antibiotics. No other costs were considered. Time spent treating and looking after diseased animals was not collected as part of the survey as it is normally considered to be part of the daily farming activities.

### Income Loss Due to Reduced Milk Production

Milk loss due to clinical LSD was estimated as the difference in the average daily milk yield in the herd before the onset of the outbreak and the daily milk yield during the outbreak (since the first affected animal showed clinical signs until the last animal recovered or died). We assumed that the difference was solely due to the disease and that the difference was the same across the outbreak period regardless of the length. None of the herds or flocks affected reported sheep or goat milk production as it is not common practice in the study area to milk these animals or commercialize their milk. Therefore, only cattle milk loss was considered. The loss of income given the drop on milk production was only estimated for those herds that reported selling milk. The price at which farmers sold their milk was not collected as part of the survey, therefore we used the milk price estimated by the local vet in the study area.

### Total Economic Losses

The total economic losses per individual herd or flock was estimated as the difference of the value of the herd before and after the outbreak plus the treatment cost and income loss due to reduced milk production.

### Statistical Analysis

Pearson's Chi squared tests (or Fisher's Exact tests where appropriate) were used to determine the strength of association between the binary outcomes of two groups. For continuous variables parametric (*t*-test or anova) or non-parametric equivalent if appropriate (Mann–Whitney *U* or Kruskal–Wallis tests) were used to compare outcomes of different groups.

The exchange rate used in the paper for cost calculations was US$1 = ₦364—valid on October 11th 2018 at www.xe.com. Analysis was performed in R 3.5.1 (25).

## Results

### Characteristics of Farms Included in the Study

**Figure 4:**
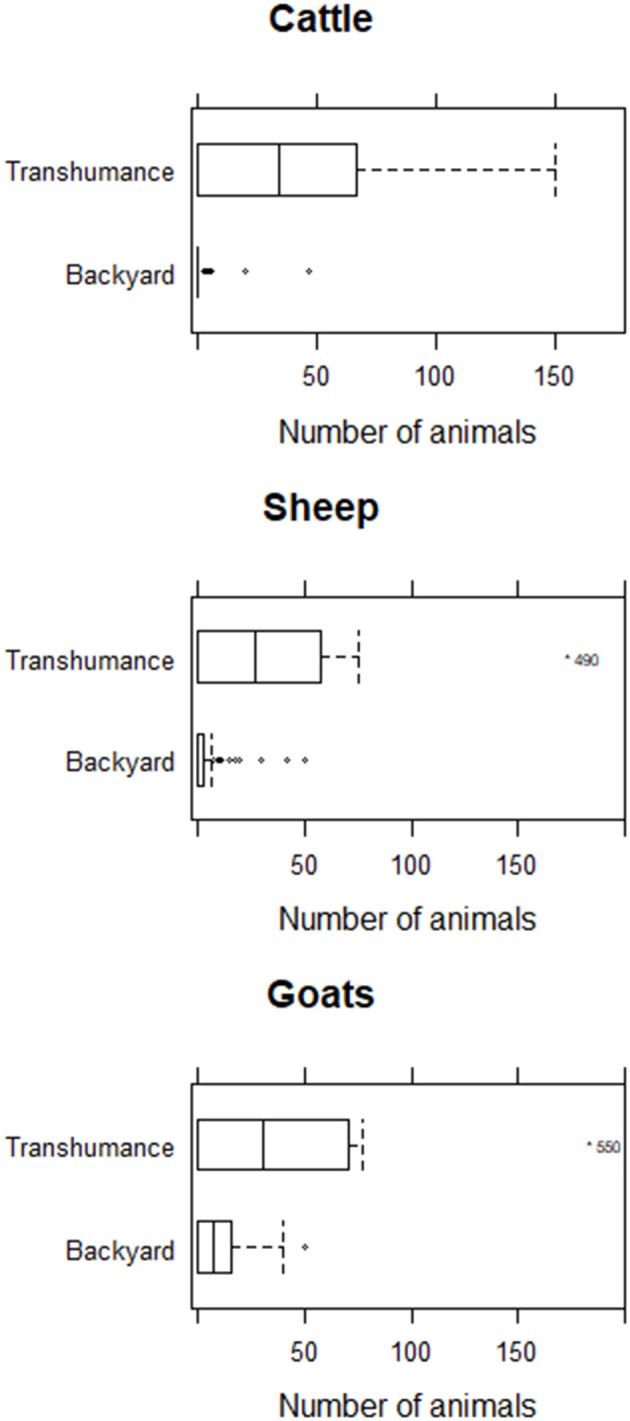
Number of animals in herds and flocks included in the study stratified by animal species.

A total of 120 affected farmers were identified and asked to take part of the study. Data were collected from 102 farmers that accepted to take part in the study. Three questionnaires were discarded either because they were incomplete (i.e., data were missing from more than half of the questionnaire) or the original questionnaire was lost. Therefore, data from 99 farmers were considered in the analysis, from 87 (88%) backyard and 12 (12%) transhumance systems. The majority of farmers came from Bauchi (*n* = 38), followed by Warji (*n* = 27), Darazo (*n* = 24), Tafawa Balewa (*n* = 8), and Alkaleri (*n* = 2). The most common reason given for keeping animals was to sell them live to generate income according to need (*n* = 85; 86%), slaughter them for different religious festivities such as weddings, end of Ramadan (sallah) and naming ceremonies (*n* = 55; 56%), sell them on a regular basis (*n* = 27; 27%), as draft animals (*n* = 18; 18%), to sell milk (*n* = 10; 10%), to commercialize their meat or consume it at home (*n* = 9; 9%), and to commercialize their milk or consume it at home (*n* = 6; 6%). Thirty-four farmers owned cattle, 49 owned sheep and 82 owned goats, with half of them (51%) keeping more than one species. Herd and flock sizes were variable and skewed to the right with transhumance farmers having larger herds and flocks than backyard farmers (Table 2 and Figure 4).

**Table 2:** Number of animals in herds and flocks included in the study stratified by animal species and local government.

**Local government**	**Production type**	**Number**	**Cattle**		**Sheep**		**Goats**	
			**Median number** **(1st−3rd qtl)**	***p***	**Median number** **(1st−3rd qtl)**	***p***	**Median number** **(1st−3rd qtl)**	***p***
All (*n* = 99)	BY	87	0 (0–0)		0 (0–3)		8 (0–16)	
	TH	12	34 (0–65)	<0.001	27 (0–55)	0.0003	31 (0–68)	0.22
**Local government**								
Alkaleri (*n* = 2)	BY	1	0^a^		30^a^		50^a^	
	TH	1	95^a^	–	51^a^	–	250^a^	–
Bauchi (*n* = 38)	BY	37	0 (0–0)		0 (0–0)		18 (12–27)	
	TH	1	18^a^	–	15^a^	–	9^a^	–
Darazo (*n* = 24)	BY	24	0 (0–0)		0 (0–1)		4 (2–6)	
	TH	0	–	–	–	–	–	–
Tafawa Balewa (*n* = 8)	BY	3	0 (0–10)		15 (8–29)		0 (0–0)	
	TH	5	0 (0–63)	0.73	45 (38–65)	0.14	65 (52–65)	0.08
Warji (*n* = 27)	BY	22	1 (0–4)		0 (0–2)		0 (0–9)	
	TH	5	50 (10–59)	0.01	0 (0–0)	0.85	0 (0–0)	0.85

BY, Back yard; TH, Transhumance;

a *Only one farm*.

*Data collected between August 2017 and January 2018 in Bauchi State, Nigeria*.

### Disease Impact

The median outbreak duration was 39 days (min 7–max 251 days). Half of the farmers interviewed (*n* = 50) reported having only goats affected; 16 (16.2%) had only cattle affected and 15 (15.2%) only sheep affected; while 12 (12.1%) had sheep and goats affected. Five farmers (5%) reported having animals from all three species showing clinical signs concurrently and 1 (1%) had cattle and sheep affected. The median outbreak duration was longer in herds or flocks when more than one species was affected compared to those that had only one species affected, however confidence intervals overlap and these differences were not statistical significant (*p* = 0.17). Similarly, median duration of outbreaks was longer in transhumance herds and flocks (57 days) than in backyard herds (38 days) but this difference was not significant (*p* = 0.13). Time between the end of the outbreak and visit from the surveyor was on average 125 days (min 1; max 420 days).

Considering only outbreaks reported during 2017 (when data on outbreaks across the entire year were collected), outbreaks were reported all year round with an increase between February and April and a second peak during August, just before the end of Ramadan (at the beginning of September) (Figure S1).

The median incidence risk and fatality rate were 33% (min 7; max 100%) and 0% (min 0; max 52%) in cattle, 53% (min 11; max 100%) and 34% (min 0–max 100%) in sheep and 50% (min 18; max 100%) and 33% (min 0; max 80%) in goats, respectively. Young stock (<1 year) had a higher incidence risk and fatality rates than adult stock (≥1 year old) (Table 3). When comparing backyard vs. transhumance herds and flocks, backyard cattle herds, and sheep flocks had higher incidence risks but lower fatality rates than transhumance cattle herds and sheep flocks (Table 3 and Supplementary Material). Incidence risk and fatality rate in goats were similar among both production systems.

**Table 3:** Median lumpy skin disease, sheep pox, and goat pox incidence risk and fatality rate in affected farms in Bauchi State, Nigeria stratify by species and production system.

**Production system**		**Cattle**					**Sheep**					**Goats**				
	**Age category**	***N***	**Incidence risk** **median** **(1st−3rd qtl)**	***p***	**Fatality rate** **median** **(1st−3rd qtl)**	***p***	***N***	**Incidence risk** **median** **(1st−3rd qtl)**	***p***	**Fatality rate** **median** **(1st−3rd qtl)**	***p***	***N***	**Incidence risk** **median** **(1st−3rd qtl)**	***p***	**Fatality rate** **median** **(1st−3rd qtl)**	***p***
All (*n* = 99)	Young stock	7	57.1 (40.2–73.8)		33.3 (7.1–49.2)		25	53.9 (44.7–70.6)		50.0 (38.1–73.2)		58	60.0 (46.6–71.1)		50.0 (28.6–66.7)	
	Adults	21	25.0 (18.2–33.3)	0.04	0 (0–0)	0.08	32	50.0 (31.9–66.7)	0.75	0 (0–27.9)	<0.001	61	50.0 (40.0–66.7)	0.55	0 (0–0)	<0.001
Backyard (*n* = 87)	Young stock	3	87.5 (64.6–93.8)		14.3 (7.1–57.1)		17	61.5 (47.1–70.6)		50.0 (36.7–69.1)		51	60.0 (47.7–66.7)		50.0 (28.6–66.7)	
	Adults	13	33.3 (25.0–50.0)	0.18	–	–	24	57.5 (50.0–69.9)	0.56	0 (0–20.5)	<0.001	54	50.0 (40.0–66.7)	0.79	0 (0–0)	<0.001
Transhumance (*n* = 12)	Young stock	4	47.93 (35.3–57.9)		36.7 (25.0–44.6)		8	50.3 (43.5–64.2)		60.7 (44.1–75.9)		7	71.4 (51.3–77.5)		47.6 (31.9–61.7)	
	Adults	8	12.1 (9.5–19.2)	0.02	0 (0–30.8)	0.38	8	25.9 (20.0–33.5)	0.03	24.9 (12.5–89.3)	0.42	7	56.6 (44.0–60.8)	0.12	21.1 (0–22.5)	0.02

*Young stock <1 year old; Adults≥ 1 year old; qtl, quartile*.

*Data collected between August 2017 and January 2018*.

Farmers reported a median drop in milk production of 65% when cows were clinically affected and 35% after they recovered, while the median weight lost in cattle was 10% during the acute phase of the disease and sheep and goats lost 15% of their live weight (Table 4).

**Table 4:** Median percentage of milk drop and weight lost reported by farmers in farms affected with lumpy skin disease, sheep pox, and goat pox in Bauchi State, Nigeria stratify by species.

**Species**	**Median percentage of milk drop during outbreak (1st−3rd qtl)^**a**^**	**Median percentage of milk drop after outbreak (1st−3rd qtl)^**a**^**	**Median percentage of weight lost (1st−3rd qtl)**
Cattle	64.6 (57.1–68.8)	35.0 (30.6–41.5)	10.0 (10.0–31.3)^b^
Sheep			15.0 (15.0–20.0)^c^
Goat			15.0 (10.0–17.0)^d^

qtl, quartile;

a Only 4 farmers responded;

b Based on responses from 20 farmers;

c Based on responses from 32 farmers;

d *Based on responses from 66 farmers*.

*Data collected between August 2017 and January 2018*.

### Management and Coping Strategies

Almost all farmers (*n* = 93; 94%) treated affected animals with antibiotics, spending a median of ₦714.29 (US$1.96) (min ₦71.43; max ₦10,000) per herd per day and treated individual animals for a median of 7 days (min 3; max 30 days). No information was collected regarding time when treatment started in relation to the outbreak onset. Similar treatment duration was reported among both production systems. Transhumance farmers spent more in treatment per day (median ₦2,628.6; US$7.2) than backyard farmers (median 676.8; US$1.9) (*p* < 0.001). Assuming all affected animals were treated equally, the median cost of treatment per affected animal was ₦750; US$2.1 and was slightly higher for transhumance farmers (₦973.3; US$2.7) than for backyard farmers (₦720.8; US$2.0) (*p* = 0.68).

Slaughtering or selling affected animals at relatively lower prices were common coping strategies. Transhumance farmers sold and slaughtered a higher proportion of animals affected than backyard farmers. Farmers, regardless of the production system, reported selling live animals for less than they would have been sold for if the animal was healthy, median percentage lost in cattle was 46.6%, while for sheep and goats this was 57.9 and 56.8% less, respectively. Median live animal prices were higher in livestock markets than if animals were sold direct to butchers for both healthy and affected animals (Table 5), but these differences were not statistically significant. Furthermore, farmers got more cash when selling live sick animals than when slaughtering them on farm and selling the meat (Tables 5, 6).

**Table 5:** Differences in prices of live animals sold (with and without the clinical signs of lumpy skin disease, sheep pox, or goat pox) reported by transhumance and backyard farmers in Bauchi State, Nigeria.

**Specie**	**Place sold**	**Production type**	**Median number of animals sold**	**Median number of affected animals^**a**^**	**Median number of affected animals overall (1st−3rd qtl)**	**Median price per affected animal sold** **(1st−3rd qtl) ₦**	**Median potential price per animal sold^**b**^** **(1st−3rd qtl) ₦**	**Median percentage lost per affected animal sold**
Cattle	Livestock market	BY (*n* = 3)	5	10	2 (1–10)	45,000 (33,000–60,000)	84,286 (80,143–87,643)	47
		TH (*n* = 5)	7	15		30,857 (30,000–42,000)	71,429 (67,500–80,000)	63
	Butchers	BY (*n* = 0)	0	0		–	–	–
		TH (*n* = 1)	2^†^	6^†^		35,000^†^	60,000^†^	42
Sheep	Livestock market	BY (*n* = 4)	4	6	6 (3–12)	4,000 (3,875–4,250)	8,700 (6,750–11,050)	47
		TH (*n* = 7)	12	21		3,857 (2,750–4,708)	9,083 (6,500–12,262)	60
	Butcher	BY (*n* = 11)	2	7		3,500 (2,750–4,000)	8,000 (6,917–10,000)	58
		TH (*n* = 1)	15^†^	190^†^		5,000^†^	9,000^†^	44
Goats	Livestock market	BY (*n* = 20)	3	8	7 (3–15)	3,000 (3,000–3,625)	6,679 (5,900–7,625)	53
		TH (*n* = 5)	13	47		2,617 (2,000–4,154)	6,667 (6,000–9,615)	60
	Butcher	BY (*n* = 28)	3	7		2,786 (2,375–3,000)	6,143 (5,625–9,250)	58
		TH (*n* = 2)	14	141		3,931 (3,665–4,196)	7,433 (7,217–7,650)	47

a For those farmers that reported selling animals;

b *Price the animal would have sold if it hadn't had the disease; Only one price given*.

*Data collected between August 2017 and January 2018*.

**Table 6:** Differences on prices of animals sold for slaughter (with and without the disease) reported by transhumance and backyard farmers in Bauchi State, Nigeria.

**Specie**	**Median percentage of affected animals slaughtered (1st−3rd qtl)**	**Median price per affected animal slaughtered** **(1st−3rd qtl) ₦**	**Median potential price per animal slaughtered^**a**^** **(1st−3rd qtl) ₦**	**Median percentage lost per affected animal slaughtered**	**Mean percentage sold^**b**^**	**Mean percentage kept for home consumption^**c**^**
Cattle	0 (0–0)	16,000 (15,000–23,000)	40,000 (35,000–70,000)	60.00	87.50	12.50
Sheep	2 (0–28)	3,333 (2,500–4,000)	7,750 (5,188–8,500)	57.50	77.73	22.20
Goats	17 (0–29)	2,450 (2,000–3,000)	5,000 (4,667–6,200)	59.17	90.28	4.86

a Price the animal would have sold if it hadn't had the disease;

b Median was 100% for all species;

c *Median was 0% for all species*.

*Data collected between August 2017 and January 2018*.

The median time between the onset of clinical signs and selling the affected animals was 11 days for cattle, 7 days for sheep and 7 for goats with no statistical difference between production systems. For all species the minimum time for selling animals was 2 days and the maximum 14 days after the onset of clinical signs.

### Total Economic Losses During the Outbreak

Economic losses during the outbreak are presented in Tables 7, 8. The median overall losses was ₦45,000 (US$123.6) ranging from ₦3,500 (US$9.6) to ₦2,307,921 (US$6,340). Losses were significantly higher (*p* < 0.001) in transhumance farmers compared to backyard farmers (Table 7). The median losses were higher, regardless of the production system, when the three animal species were affected followed by when both small ruminant species were affected, although the number of farmers in each category was small (Table 8).

**Table 7:** Economic losses due to lumpy skin disease, sheep pox, and goat pox outbreaks in subsistence farmers in Bauchi Nigeria.

	**Overall** **median (min–max)**	**Backyard** **median (min–max)**	**Transhumance** **median (min–max)**
Value of the herd before the outbreak	120,000 (13,140–11,400,000)	100,000 (13,140–3,961,429)	3,020,000 (660,000–11,400,000)
Value of the herd after the outbreak	72,000 (8,000–10,990,000)	65,000 (8,000–3,490,000)	2,930,000 (309,500–10,990,000)
Value of the herd after the outbreak (without cash)	57,990 (7,000–10,990,000)	52,000 (7,000–3,175,000)	2,852,500 (260,500–10,990,000)
• Loss due to mortality	14,000 (0–1,295,714)	14,000 (0–260,000)	143,500 (0–1,295,714)
• Value from affected animals kept	3,500 (0–1,259,539)	3,000 (0–225,000)	41,908 (0–1,259,539)
• Value from animals not affected	52,000 (0–10,640,000)	48,000 (0–2,950,000)	2,740,000 (237,000–10,640,000)
• Money from selling live animals affected	6,000 (0–484,000)	5,000 (0–315,000)	101,500 (0–484,000)
• Money from selling meat from affected animals	0 (0–125,000)	0 (0–45,000)	30,250 (0–125,000)
Treatment cost	4,500 (0–150,000)	3,500 (0–31,000)	20,500 (2,500–150,000)
Money loss from drop on milk production	0 (0–413,250)	0 (0–162,500)	0 (0–413,250)
Total loss ₦	45,000 (3,500–2,307,921)	42,700 (3,500–756,500)	501,317 (43,500–2,307,921)
US$	123.6 (9.6–6,340.4)	117.3 (9.6–2,078.3)	1,377.2 (119.5–6,340.4)
% of the value of the herd loss	38 (2**–**76)	42 (11**–**76)	24 (2**–**67)
Total loss—without cash ₦	62,000 (3,570–2,836,921)	49,000 (3,570–906,500)	653,317 (43,500–2,836,921)
from animals & meat sold US$	170.3 (9.8–7,793.7)	134.6 (9.8–2,490.4)	1,794.8 (119.5–7,793.7)
% of the value of the herd loss	46 (5**–**95)	50 (11**–**95)	28 (5**–**73)

**Table 8:** Total losses due to lumpy skin disease, sheep pox, and goat pox per herd by production system and species affected.

	**Only cattle affected** ***n* = 16**	**Only sheep affected** ***n* = 15**	**Only goats affected** ***n* = 50**	**Cattle and sheep affected** ***n* = 1**	**Goats and sheep affected** ***n* = 12**	**Cattle sheep and goats affected** ***n* = 5**
Num. backyard herds (*n* = 87)	*n* = 12	*n* = 15	*n* = 50	*n* = 0	*n* = 8	*n* = 2
Total loss^a^	63,600 (22,000–756,500)	24,000 (5,000–241,500)	30,250 (3,500–176,857)	–	70,667 (18,000–341,500)	92,210 (69,500–114,920)
Total loss US$^a^	174.7 (60.4–2,078.3)	65.9 (13.7–663.5)	83.1 (9.6–485.9)	–	194.1 (49.5–938.2)	253.3 (190.9–315.7)
Total loss ₦^a^^,^^b^	83,950 (42,200–906,500)	29,500 (5,000–288,500)	42,750 (3,570–221,857)	–	101,167 (20,000–380,500)	109,960 (88,000–131,920)
Total loss US$^a^^,^^b^	230.6 (115.9–2,490.4)	81.0 (13.7–792.6)	117.4 (9.8–609.5)	–	277.9 (54.9–1,045.33)	302.1 (241.8–362.4)
Num. transhumance herds (*n* = 12)	*n* = 4	*n* = 0	*n* = 0	*n* = 1	*n* = 4	*n* = 3
Total loss ₦^a^	105,500 (43,500–838,250)	–	–	1,529,964	501,317 (245,500–2,037,662)	2,047,864 (224,667–2,307,921)
Total loss US$ ^a^	289.8 (119.5–2,302.9)	–	–	4,203	1,377.2 (674.5–5,598.0)	5,626.0 (617.2–6340.4)
Total loss ₦^a^^,^^b^	183,000 (43,500–838,250)	–	–	1,877,964	653,317 (296,500–2,227,662)	2,387,864 (355,667–2,836,921)
Total loss US$^a^^,^^b^	502.7 (119.5–2,302.9)	–	–	5,159.0	1,794.8 (814.6–6,119.9)	6,560.1 (977.1–7,793.7)

a *Median (min–max)*.

b *Without considering money obtained from animals sold (live or as meat) as part of the value of the herd or flock after the outbreak*.

The median percentage loss on the value of the herd or flock was 33%, with backyard farmers losing a higher proportion of the value (median 36; min 9, max 70%) than transhumance farmers (median 20; min 1; max 64%) (*p* = 0.03).

Money obtained from selling live animals with disease made 6.1% of the value of the herd before the outbreak, with an important difference across and between production systems (backyard farmers: median 7%; min 0, max 41%, and transhumance farmers: median 3%; minimum 0; maximum 12%) (Table S3). When money obtained from selling or slaughter diseased animals was not considered as part of the value of the herd after the outbreak, the median overall loss went up to ₦62,000 (US$170), ranging from ₦3,570 (US$9.8) to ₦2,836,921 (US$7,794) (Table 7 and Table S3). This would be the case if money obtained by selling animals is not used to purchase animals in order to re-stock the herd of flock.

### Long Term Impact

The majority of farmers (*n* = 71; 71.7%) had not replaced animals (sold or slaughtered) at the time of interview (66 out of 87 backyard farmers and 5 out of 12 transhumance farmers). One farmer reported replacing all animals and the remaining (*n* = 27; 27.3%) replaced only part of the animals lost (20 out of 87 backyard farmers and 7 out of 12 transhumance farmers). The main reasons given for not replacing lost animals were lack of resources and concern of further disease outbreaks. Livestock markets were the most common place to buy animals for both backyard and transhumance farmers. Out of 75 farmers that provided an answer on the place where the last animal(s) was purchased, 64 (85.3%) reported it was from a livestock market, followed by neighbors (*n* = 11; 14.7%—all backyard farmers) and middleman (*n* = 3; 4.0%—all backyard farmers) with some farmers buying from more than one source. The median prices farmers reported for purchased animals were ₦85,000 (US$233.5) for cattle, ₦8,000 (US$22.0) for sheep and ₦5,250 (US$14.4) for goats, which was 63.7%, 61.1% and 50.0% higher than the payment they received for selling their diseased animals.

## Discussion

This study quantifies the direct production losses and additional costs that subsistence farmers incur as a consequence of the three CPPV diseases in Nigeria, and identifies coping strategies that might be playing a role in virus transmission. This work builds on previous studies investigating the impact of CPPV disease on livestock in different settings (1, 2, 9, 26). The results from this study suggest that LSD, SP, and GP have an immediate as well as potentially long lasting impacts on subsistence farmers' livelihoods with important differences between production systems.

Slaughtering or selling affected animals at a lower price than their full market value were common coping strategies reported in this study. These practices not only have an obvious immediate negative financial impact on the farmers, but also in the long term by changing the herd structure. Seven out of every 10 farmers reported they had not replaced animals that died, were sold or slaughtered as a consequence of the outbreak. Price reduction on affected cattle has been reported in India and Ethiopia (1, 2). In comparison to the current study, a similar price reduction was reported in India (farmers reported losing around 50% of the market value when selling affected cattle), while in Ethiopia the reported loss was due to a reduction in beef production. No previous reports exist on sheep and goats. A limitation of this and previous studies is that price reductions were based on retrospective farmers estimates. Although informal conversations with livestock buyers and butchers in Bauchi suggested that price reduction reported by farmers is within a realistic range, this should be systematically recorded in future studies, ideally at the time the transaction happens, in order to make an objective comparison. Treating animals with antibiotics was also a common control measure, representing an additional cost for farmers. Antibiotic treatment is often reported as a control measure to reduce secondary infections in cattle (3, 26). Important differences between production systems were identified in this study. Transhumance farmers sold and slaughtered, on average, a higher proportion of animals affected and the money obtained from selling animals represented less of the value of the herd after the outbreak than for backyard farmers yet the percentage loss of the value of the herd or flock is higher in backyard farmers. Differences in the decision making process might be related to herd and flock sizes, management practices, or access to markets. Transhumance farmers may be able to sell a high proportion of animals and still have enough animals left to meet their basic requirements. In contrast, for backyard farmers with smaller herds and flocks, selling a similar proportion of animals has a relatively higher impact potentially making it unsustainable. Driving diseased animals might slow down transition time from one grazing area to another, potentially playing a role in the decision to sell affected animals for transhumance farmers. These findings suggest that control measures to decrease virus transmission between herds could initially be directed toward transhumance farmers. Further studies should be conducted to test these hypotheses and to better understand the decision making process and its implications for controlling CPPV.

Looking at the epidemiological parameters in this study, incidence risk and fatality rate were higher in young animals compared to adults. Similar findings have been reported in other endemic settings (5, 21, 27). A potential explanation is that in endemic settings adult animals have been previously exposed to the virus and have some level of naturally acquired immunity, while in naïve populations animals across all ages are exposed for the first time and all get equally affected. Moreover, young animals are exposed for the first time when their immune system is still immature increasing mortality rate considerably in this group. When looking at differences across species, incidence risk, and fatality rates were lower for LSD than for SP and GP which is consistent with previous reports (7). However, the fatality rate parameters reported in this study for LSD are lower than those reported in Tunisia (21). The overall incidence risk and fatality rate estimates in this study might be underestimated as selling and slaughtering affected animals was a common practice, potentially reducing these two parameters at herd level. Selection bias is also possible through participating farmers being more severely affected than other farmers in the area who may not have reported disease which may be more likely if the disease incidence is lower. Comparing the different production systems, incidence risk was higher in backyard cattle herds and sheep flocks, which might reflect the different stocking densities and subsequent transmission risks. Additionally, based on the experience of the authors, it is common practice for backyard farmers to release small ruminants in the morning to communal pastures where animals mix with other herds and flocks, while keeping cattle within the farm enclosure. Conversely, transhumance farmers keep animals in extensive systems rarely mixing with other herds and flocks.

Although most of the susceptible animals to LSD, SP, and GP are kept by subsistence producers in northern Nigeria (backyard and transhumance farmers), it is important to highlight that this was an exploratory study using non-probabilistic sampling, in which only farmers affected by the disease from 5 (out of 20) local governments in Bauchi state were invited to take part and participation was voluntary. Therefore, the extent to which LSD, SP, and GP are present in the study area cannot be estimated. Moreover, the lack of official registers (of the number of animals kept by each production system and in each local government) preclude an assessment on the extent of representativeness of farmers that took part of the study among the livestock population in Bauchi State. Farmers that took part in the study might have been different or affected differently than those that did not accept to take part. Further studies using a probabilistic sampling should be conducted in order to estimate the prevalence and understand the extent of the problem.

In this study some farmers reported having more than one species affected and cases of SP and GP and LSD were observed concurrently in some farms. Some strains of SPPV and GTPV cause disease in both species, and outbreaks affecting sheep and goats simultaneously have been previously reported (28). However, LSDV is restricted to causing disease in cattle and water buffalo, therefore cases of SP, GP, and LSD in the same farm indicate the presence of multiple CPPV species, and possibly management practices which encourage a high influx or retention of pathogens.

Production parameters estimated in this study showed that milk production in cattle with LSD was reduced by 65% during the acute phase and 35% once cattle recovered from the disease, illustrating the marked and protracted negative impact of LSD. Similar results have been reported in Turkey and India (1, 4), while greater milk reduction was reported in Ethiopia although this also included commercial farms potentially increasing the magnitude of the impact (26). In addition to the reduced milk production, on average cattle lost an estimated fifth of their live weight which is similar to a report from Jordan (3). In our study, the reported weight loss was similar in sheep and goats and to the author's knowledge this is the first time that this loss has been quantified for SP and GP. It is important to note that health and productivity parameters, as well as selling prices are not normally recorded systematically by backyard or transhumance producers so this study is based on farmers' estimates. Furthermore, time between the end of the outbreak and the survey varied considerably across farmers that took part of the study, meaning recall or reported bias cannot be excluded. Nonetheless the information recorded and reported here is valuable baseline information that illustrates the negative impact and economic losses experienced by subsistence farmers and can be used in further studies or in estimating the benefits from control.

Assessment of the economic losses due to the outbreaks showed that losses varied depending the species affected regardless of the production system. They were higher in absolute terms for transhumance farmers but higher as a proportion of the value of the herd in backyard farmers. The more species affected, the higher reported impact and in the case of backyard farmers, those that had only one species affected the economic impact was higher when cattle were affected, followed by goats and sheep. It was not possible to estimate how this figure compared to the overall household income. In addition, the value of animals for subsistence producers goes beyond the purely financial benefits with live animal sales in the face of an outbreak possibly negatively impacting food access and availability within the household. The results from this study can be used as an initial assessment to inform policy makers when prioritizing control of livestock diseases and to lobby for vaccine availability in the country as an option to control the disease.

The quantitative assessment of the economic losses of CPPV diseases only included losses that were considered of crucial importance to farmers in the study area and were realistic to quantify with information collected during one visit. As a result it is likely that the impact of the disease is even higher. The effect of draft oxen on crop production due to LSD was not assessed in this study, but has been shown to have an important impact in similar production systems in Africa (2). Therefore, impacts on crop production such as through a reduced capacity to cultivate or additional costs associated with hiring an ox should be considered in future assessments.

It is clear from the results presented here that LSD, SP, and GP have a negative impact on farmers' livelihoods, but also that some of the control measures and coping strategies taken at a farm level are likely to have consequences for spreading virus and maintaining disease in the study area. There is evidence that vaccination can be an effective control measure for LSD, SP, and GP (5, 29), although the lack of CPPV vaccine available in Nigeria leaves farmers and other stakeholders along the production chain (e.g., middlemen and livestock markets managers) without this disease control option. Understanding farmers' incentives and motivations to implement certain practices is key when planning interventions and disease control programs. Farmers in Bauchi State get more cash when selling animals live than when selling meat. Furthermore, livestock prices at markets were higher than selling to butchers or middleman. These are clear incentives for selling live animals (regardless of their health status) in livestock markets. In addition, most affected animals were sold while likely infectious (the median time for selling animals, after appearance of clinical signs, was 10.5 days for cattle, 7.0 days for sheep, and 7.0 for goats), and livestock markets were also mentioned as common place to buy replacement animals. This suggests that livestock markets are potential hubs for virus transmission in the study area and at the same time a potential place to implement surveillance and control measures. Separating affected animals as well as animals coming from affected herds or flocks in livestock markets, disinfecting, and fumigation livestock markets during closing dates might help to reduce the transmission rate. In addition, improving farmers knowledge on diseases and CPPV transmission might help farmers to take informed decisions when purchasing animals. Further studies should be conducted to better understand animal movements and connections across livestock markets. In addition, different control measures focused on livestock markets should be explored and formally quantified and evaluated.

## Conclusions

This study demonstrates that LSD, SP, and GP have immediate as well as potentially long-lasting impacts on subsistence farmers' livelihoods in northeast Nigeria. We quantified the effect of CPPV disease on production parameters that have not been quantified before in sheep and goats, assessed the impact of the diseases on subsistence producers from different angles and identified potential transmission routes and areas to direct control measures.

## Data Availability Statement

The datasets generated for this study are available on request to the corresponding author.

## Author Contributions

GL and NL conceptualized the study and developed the questionnaire with input from AG, AA, and PB. AG diagnosed affected animals, coordinate the data collection (with input from AA), and did the data entry. GL verify data entry and conduct the data analysis with input from NL. PB secured funding. GL, NL, and PB drafted the manuscript. All authors reviewed the manuscript.

### Conflict of Interest

The authors declare that the research was conducted in the absence of any commercial or financial relationships that could be construed as a potential conflict of interest.
