# Supplementary material for: Epidemiological Characteristics and Economic Impact of Lumpy Skin Disease, Sheeppox and Goatpox Among Subsistence Farmers in Northeast Nigeria
Source: Front Vet Sci. 2020 Jan 29;7:8. doi: 10.3389/fvets.2020.00008 (PMC7001645; doi:10.3389/fvets.2020.00008)
Supplement: Supplementary file 1 [file Table_1.DOCX]

**Supplementary material**

***Table S1*** Median lumpy skin disease, sheep pox and goat pox incidence risk and mortality rate in affected farms in Bauchi State, Nigeria stratify by species and production system. Data collected between August 2017 and January 2018.

| **Age category** | **Production type** | **CATTLE** | | | | **SHEEP** | | | | **GOATS** | | | |
| --- | --- | --- | --- | --- | --- | --- | --- | --- | --- | --- | --- | --- | --- |
|  |  | **Incidence risk**  **Median**  **(1^st^ – 3^rd^ qtl)** | ***p*** | **Fatality rate**  **Median**  **(1^st^ – 3^rd^ qtl)** | ***p*** | **Incidence risk**  **Median**  **(1^st^ – 3^rd^ qtl)** | ***p*** | **Fatality rate**  **Median**  **(1^st^ – 3^rd^ qtl)** | ***p*** | **Incidence risk**  **Median**  **(1^st^ – 3^rd^ qtl)** | ***p*** | **Fatality rate**  **Median**  **(1^st^ – 3^rd^ qtl)** | ***p*** |
| **Overall** | |  |  |  |  |  |  |  |  |  |  |  |  |
| **All** | BY | 33.3 (25.5-50.0) |  | 0 (0-0) |  | 60.0 (45.4-70.0) |  | 27.7 (0-50.0) |  | 50.0 (39.4-65.4) |  | 30.9 (0-42.9) |  |
|  | TH | 16.1 (9.5-26.2) | 0.004 | 0 (0-36.9) | 0.21 | 35.5 (29.6-48.3) | 0.03 | 44.0 (36.5-77.7) | 0.07 | 61.5 (51.7-66.5) | 0.11 | 33.3 (25.7-37.2) | 0.34 |
| Young stock | BY | 87.5 (64.6-93.8) |  | 14.3 (7.1-57.1) |  | 61.5 (47.1-70.6) |  | 50.0 (36.7-69.1) |  | 60.0 (47.7-66.7) |  | 50.0 (28.6-66.7) |  |
|  | TH | 47.9 (35.3-57.9) | 0.21 | 36.7 (25.0-44.6) | 0.88 | 50.3 (43.5-64.2) | 0.90 | 60.7 (44.1-75.9) | 0.48 | 71.4 (51.3-77.5) | 0.17 | 47.6 (31.9-61.7) | 0.63 |
| Adults | BY | 33.3 (25.0-50.0) |  | - |  | 57.5 (50.0-69.9) |  | 0 (0-20.5) |  | 50.0 (40.0-66.7) |  | 0 (0-0) |  |
|  | TH | 12.1 (9.5-19.2) | 0.003 | 0 (0-30.8) | 0.12 | 25.9 (20.0-33.5) | <0.001 | 24.9 (12.5-89.3) | 0.11 | 56.6 (44.0-60.8) | 0.89 | 21.1 (0-22.5) | 0.25 |

**Table S2** Prices subsistence farmers reported selling live animals with Capripox diseases (affected animals) and potential prices if the same animal was healthy in Bauchi, Nigeria.

|  |  | **Price ₦** | |  |
| --- | --- | --- | --- | --- |
|  | **Overall**  Median (min-max) | **Backyard** (n=87)  Median (min-max) | **Transhumance** (n=12)  Median (min-max) | **P value** |
| **Affected cattle** | 35,000 (21,000 - 75,000) | 45,000 (21,000 - 7,500) | 32,929 (21,429 – 42,500) | 0.44 |
| **Affected sheep** | 4,000 (500 - 7,000) | 4,000 (967 - 7,000) | 4,262 (500 - 5000) | 0.74 |
| **Affected goats** | 3,000 (1,500 - 5,500) | 3,000 (1,500 - 5,500) | 3,400 (2,000 - 5,000) | 0.42 |
| **Healthy cattle** | 76,000 (50,000 - 91,000) | 84,286 (76,000 - 91,000) | 69,464 (50,000 - 85,714) | 0.12 |
| **Healthy sheep** | 8,000 (3,333 - 15,000) | 8,000 (4,846 - 13,000) | 9,042 (3,333-15,000) | 0.56 |
| **Healthy goats** | 6,571 (3,500 – 15,000) | 6,393 (3,500-15,000) | 7,000 (5,000-12,000) | 0.44 |
|  |  |  |  |  |

***Table S3*** Percentage losses from the total value of the herd before the outbreak in each of the categories considered to estimate the value of the herd after an outbreak of Capripox diseases in subsistence farmers in Bauchi, Nigeria.

|  | **Overall**  **Median (min – max)** | **Backyard**  **Median (min – max)** | **Transhumance**  **Median (min – max)** |
| --- | --- | --- | --- |
| % loss due to mortality | 11.7 (0- 40.7) | 12.0 (0 – 40.7) | 10.2 (0 – 24.6) |
| % loss due to affected animals sold as live animals | 6.1 (0-41.2) | 6.7 (0- 41.2) | 3.2 (0-12.4) |
| % loss due to affected animals slaughtered and meat sold | 0 (0- 13.6) | 0 (0- 13.6) | 0.8 (0-6.8) |
| % loss due to decrease of value in affected animals kept | 3.9 (0- 50) | 4.2 (0- 50) | 3.2 (0- 15.2) |

**Figure**





**Figure S1** Number of outbreaks that started in each month during 2017 among farmers interviewed in Bauchi Nigeria.
